# Rapid On-Site Detection of *Lacticaseibacillus paracasei* Using a Portable MIRA-CRISPR/Cas12a Naked-Eye Fluorescence Assay

**DOI:** 10.3390/foods15152709

**Published:** 2026-07-31

**Authors:** Kai Liao, Yang Zhang, Xiaosu Xiong, Zhonglu She, Yufeng Wang, Zihong Ye, Zefeng Shan, Yuxin Li, Xiaoran Jia, Xiaojun Zhu, Feng Xue, Jie Zou, Xiaoqiang Zhang, Wei Chen

**Affiliations:** 1 Key Laboratory of Food Contact Materials Safety, State Administration for Market Regulation, Jiangsu Product Quality Testing and Inspection Institute, Nanjing 210007, China; 2 Key Laboratory of Microbiological Metrology, Measurement & Bio-Product Quality Security, State Administration for Market Regulation, College of Life Sciences, China Jiliang University, Hangzhou 310018, China; 3 Taizhou Institute of Agricultural Science, Jiangsu Academy of Agricultural Sciences, Taizhou 225300, China; 4 MOE Joint International Research Laboratory of Animal Health and Food Safety, College of Veterinary Medicine, Nanjing Agricultural University, Nanjing 210095, China; 5 School of Food and Biological Engineering, Hefei University of Technology, Hefei 230009, China

**Keywords:** *Lacticaseibacillus paracasei*, MIRA-CRISPR/Cas12a, fluorescence visualization, on-site testing

## Abstract

Probiotics play an important role in maintaining human gastrointestinal health and immune function, driving a rapidly expanding global market for probiotic-fortified foods. *Lacticaseibacillus paracasei* is one of the most widely applied probiotic strains in functional foods and dairy formulations, with well-documented health-promoting properties. For probiotic foods, authenticity requires rigorous verification for efficacy and compliance, which depends on rapid strain-specific identification. However, conventional culture-based methods are time-consuming and cannot distinguish closely related *Lacticaseibacillus* species, while PCR-based assays require expensive thermal cyclers and trained personnel, barring deployment in resource-limited settings. Here, we report the development and validation of a MIRA-CRISPR/Cas12a fluorescence assay for rapid, species-specific, and highly sensitive on-site detection of *Lacticaseibacillus paracasei*. This integrated workflow combines 8 min rapid crude DNA extraction, 30 min isothermal MIRA pre-amplification, and 20 min CRISPR/Cas12a signal amplification with naked-eye fluorescence readout under blue light excitation, and requires only miniaturized portable equipment. The assay achieved a reliable limit of detection of 100 CFU/mL in milk-matrix spiked samples, showing no cross-reactivity across 17 tested lactic acid bacteria strains. In a validation set of 48 food samples, our method demonstrated 100% concordance with the gold-standard species-specific qPCR assay. This field-deployable method is appropriate for on-site quality control in probiotic manufacturing, label compliance verification, and frontline market regulatory inspections.

## 1. Introduction

*Lacticaseibacillus paracasei* (*L. paracasei*) is a Gram-positive, facultatively heterofermentative lactic acid bacterium [1,2]. This organism confers multiple health benefits, including improving gut function, boosting immune response, and suppressing pathogens [2,3,4]. Given these advantages, *L. paracasei* is among the most widely used probiotic strains in functional foods, dairy products, and dietary supplements [5,6].

Accurate species-level identification of *L. paracasei* in probiotic products is a fundamental requirement for quality assurance, regulatory compliance, and consumer safety [7]. Conventional culture-based identification methods that rely on colony morphology, Gram staining, and biochemical tests typically take 48–72 h and fail to distinguish closely related *Lacticaseibacillus* species [8]. While whole genome sequencing offers reliable identification, it demands 48–96 h, specialized sequencing equipment, and bioinformatics expertise, making it unsuitable for high-throughput routine quality control [9]. By contrast, qPCR has become the mainstream laboratory method for the identification of *L. paracasei* due to its rapid turnaround and high accuracy [10]. However, qPCR depends on expensive thermal cyclers and trained personnel, precluding deployment in resource-limited settings, and its 10^2^–10^3^ CFU/mL detection limit fails to capture low-level contamination [11,12].

Isothermal amplification technologies operate at a constant temperature and are frequently coupled with CRISPR systems for highly sensitive and specific nucleic acid detection [13]. Among these, loop-mediated isothermal amplification (LAMP) is typically combined with Cas12b, as both systems exhibit optimal activity at 60–65 °C; however, LAMP requires multiple primers (typically 4–6), which increases the risk of non-specific amplification, and its amplification efficiency is inferior to that of recombinase polymerase amplification (RPA) [14,15]. In contrast, RPA or recombinase-aided amplification (RAA) is commonly integrated with Cas12a, with both systems optimally operating at 37–42 °C and requiring only a single primer pair; consequently, detection methods based on this combination have been most widely developed [15,16]. Multienzyme Isothermal Rapid Amplification (MIRA), an isothermal technique analogous to RPA, utilizes *Streptomyces*-derived thermostable RecA recombinase instead of the T4 bacteriophage-derived UvsX recombinase employed in RPA, thereby circumventing patent restrictions associated with commercial RPA kits [17,18]. This enzymatic distinction endows MIRA with superior tolerance to complex food matrices and reduced non-specific amplification in crude extracts [18,19].

To date, there remains a critical lack of on-site visual detection methods for *L. paracasei* that simultaneously meet four core requirements: high species specificity to differentiate closely related members of the *L. casei* group, ultra-high sensitivity, compatibility with simplified sample pretreatment, and adaptability to miniaturized portable instrumentation. This unaddressed technical gap has severely restricted frontline regulatory oversight of commercial probiotic products, creating an urgent demand for robust, field-deployable detection tools. Herein, we developed and validated a MIRA-CRISPR/Cas12a fluorescence assay for rapid, species-specific detection of *L. paracasei*, which provides an operationally straightforward, robust, and field-ready analytical platform for on-site probiotic quality control and frontline regulatory inspections.

## 2. Materials and Methods

### 2.1. Bacterial Strains and Culture Conditions

A panel of 23 bacterial strains, commonly used in probiotic products and spanning 6 genera, was employed. Type strains comprised *Lacticaseibacillus paracasei* CICC 20286, *Lacticaseibacillus casei* CICC 6117, *Lacticaseibacillus rhamnosus* CICC 6001, *Lactobacillus helveticus* CICC 20243, *Lactiplantibacillus plantarum* CICC 10345, *Lactococcus lactis* subsp. *lactis* CICC 20400, *Pediococcus pentosaceus* CICC 22228, and *Bifidobacterium animalis* subsp. *lactis* CICC 21709, all obtained from the China Center of Industrial Culture Collection (CICC, Beijing, China). Commercial probiotic strains preserved in our laboratory included *L. paracasei* GM080, GMNL-33, LcS, K56, and LC01; *L. casei* BD-8632, B63, B240, ATCC 393, and LC89; and *L. rhamnosus* GG (LGG^®^), HN001, MP108, GR-1, and ATCC 53103.

All strains were anaerobically cultured in De Man–Rogosa–Sharpe (MRS) broth (HuanKai Microbial, Guangzhou, China; Cat#027312) at 37 °C for 24–48 h. Bacterial concentrations were quantified by standard plate counting: serial 10-fold dilutions of bacterial suspensions were prepared with sterile phosphate-buffered saline (PBS, pH 7.4), and 100 μL of each dilution was spread on MRS agar plates (HuanKai Microbial, Guangzhou, China), which were incubated anaerobically at 37 °C for 48 h before colony enumeration.

### 2.2. DNA Extraction

Rapid crude extraction (RCE) was performed using a commercial kit (AMP-Future Biotech, Changzhou, China; Cat#WLD8201-ES), which is optimized for MIRA isothermal amplification and exhibits broad compatibility with cell culture media, animal tissues, and food matrices. Bacterial lysis was achieved via drastic pH shock followed by rapid neutralization, yielding a stable crude DNA extract without the need for repeated purification. The modified procedure was as follows: 100 μL of fresh logarithmic-phase bacterial culture suspension or LAB-spiked food homogenate was mechanically lysed with 0.1 mm diameter sterile glass beads (Sigma-Aldrich, St. Louis, MO, USA) by vigorous vortexing at maximum speed (3000 r/min, 4.8 mm orbital diameter) for 2 min on an MX-E vortex mixer (DLAB Scientific, Beijing, China). Subsequently, 16 μL of Reagent A and 4 μL of Reagent B were added, and the mixture was incubated at 40 °C for 5 min. The lysate was then centrifuged at 6708× *g* for 1 min in a D1012U mini centrifuge (DLAB Scientific, Beijing, China). The resulting supernatant was collected.

For comparative validation, conventional genomic extraction (CGE) was performed using a genomic DNA extraction kit (Sangon Biotech, Shanghai, China; Cat#B518225) as the reference method. This procedure employs enzymatic lysis with lysozyme followed by heat-mediated alkaline digestion and isopropanol-based purification; *Lactobacillus helveticus* required additional mutanolysin (200 U/mL, Cat# M9901, Sigma-Aldrich, St. Louis, MO, USA) treatment. Bacterial cells were harvested by centrifugation, pretreated with lysozyme (20 mg/mL) at 37 °C, and subsequently lysed in Buffer Digestion at 65 °C. After protein precipitation and isopropanol-mediated DNA recovery, purified DNA was dissolved in TE Buffer (pH 8.0).

DNA purity and concentrations were measured spectrophotometrically using a Spark multi-mode microplate reader (Tecan Trading Co., Ltd., Shanghai, China). Extraction integrity was validated by universal bacterial *16S rRNA* gene PCR using primers 16SF/1495R (Table 1) with subsequent agarose gel electrophoresis to confirm the expected amplicon size.

### 2.3. Primer and crRNA Design

The species-specific target region was identified through comparative genomic analysis using SnapGene 8.2 software (Dotmatics, Boston, MA, USA). The *rpsM* gene, a conserved single-copy housekeeping gene encoding the 30S ribosomal protein S13, was selected as the amplification target. MIRA primers were designed according to established criteria: length of 30–35 bp, GC content of 30–70%, and absence of predicted primer dimers, hairpin structures, or cross-reactivity with other lactic acid bacteria (LAB) genomes. The crRNA was designed to target the *L. paracasei*-specific sequence within the MIRA amplicon. It consists of a universal scaffold sequence and a 23-nt specific spacer sequence complementary to the region downstream of the 5′-TTTC-3′ protospacer adjacent motif (PAM). All oligonucleotides, including MIRA primers, crRNA, and 6-carboxyfluorescein (FAM)-labeled single-stranded DNA (ssDNA) fluorescent reporter, were synthesized and HPLC-purified by Sangon Biotech Co., Ltd. (Shanghai, China). Sequences are provided in Table 1.

### 2.4. MIRA Isothermal Amplification

MIRA isothermal pre-amplification was performed using the commercial AMP-Future MIRA Basic Kit (AMP-Future Biotech Co., Ltd., Changzhou, China; Cat#MIRA001) according to the manufacturer’s protocol with minor system optimizations. Each 50 μL reaction contained: 29.4 μL Buffer A, 2 μL forward primer (MIRA-F, 10 μM), 2 μL reverse primer (MIRA-R, 10 μM), 5 μL of genomic DNA template, and 9.1 μL of nuclease-free water. The reaction was initiated by adding 2.5 μL Buffer B and immediately incubated at 37 °C for 30 min in a dry heat block (Eppendorf, Hamburg, Germany). Filter-tipped pipettes were used throughout to prevent cross-contamination. All post-amplification tube opening and sample transfer operations were performed in a dedicated post-amplification area to minimize aerosol contamination risks, given the high amplicon concentration generated.

### 2.5. Optimization of CRISPR Reaction Conditions

Systematic single-factor optimization was performed to maximize Cas12a trans-cleavage activity. First, a gradient of Cas12a:crRNA molar ratios (1:5, 2:5, 4:5, 8:5, 16:5, 32:5, and 64:5) was tested to identify the optimal condition for functional ribonucleoprotein (RNP) complex assembly. Second, under the optimized Cas12a:crRNA ratio, a gradient of Cas12a:crRNA:ssDNA molar ratios (4:5:6.25, 4:5:12.5, 4:5:25, 4:5:50, 4:5:100, 4:5:200, and 4:5:400) was evaluated. Third, based on the optimal ratio, working concentrations were tested across a Cas12a gradient (4, 8, 16, 32, 64, and 128 nM). All CRISPR reactions contained 1× NEBuffer 2.1 (NEB, Ipswich, MA, USA), 5 μL MIRA product, and nuclease-free water to a final volume of 100 μL. Fluorescence signals were recorded every 1 min for 45 min using a Spark multi-mode microplate reader (Tecan Trading Co., Ltd., Shanghai, China) with excitation at 485 nm and emission at 535 nm. All reactions were performed at a constant 37 °C, consistent with the MIRA amplification temperature. The time to reach fluorescence plateau and end-point fluorescence intensity were recorded as evaluation indicators.

### 2.6. Specificity Evaluation of the Assay

The analytical specificity of the established MIRA-CRISPR/Cas12a fluorescence assay was assessed using 23 LAB strains spanning 6 common genera approved for food use (detailed in Section 2.1). Fresh cultures of each strain were enumerated by viable plate counting and adjusted to a uniform concentration of 1 × 10^7^ CFU/mL. Genomic DNA was extracted from each strain, as described in Section 2.2, and tested in parallel under the optimized assay conditions. Fluorescence signals were visualized using a 470 nm blue light transilluminator (Puribo Instruments, Hangzhou, China). A positive result was defined as the presence of green fluorescence detectable by the naked eye, while the absence of visible fluorescence was designated as a negative result.

### 2.7. Sensitivity Evaluation of the Assay

The limit of detection (LOD) of the established MIRA-CRISPR/Cas12a fluorescence assay was determined in a milk matrix using 10-fold serial dilutions of *L. paracasei* CICC 20286. Overnight cultures of the strain were enumerated via viable plate counting, spiked into sterile milk, and serially diluted therein to yield final concentrations ranging from 1 × 10^0^ to 1 × 10^10^ CFU/mL. Genomic DNA was extracted from each milk-borne dilution and tested in parallel using two detection strategies to validate the signal enhancement effect of MIRA pre-amplification: (1) standalone CRISPR/Cas12a fluorescence detection without MIRA pre-amplification; (2) MIRA-CRISPR/Cas12a fluorescence detection with the established assay. Fluorescence signals were visualized and recorded using a 470 nm blue light transilluminator for all tests. The reliable LOD was defined as the lowest bacterial concentration yielding consistent positive signal across all independent replicates (9/9, 3 biological × 3 technical). The positivity criteria and quantitative image analysis procedures were consistent with those described in Section 2.6. For concentrations below the reliable LOD, sporadic positive signals attributable to stochastic sampling effects were analyzed using Poisson distribution theory.

### 2.8. Detection of Real Food Samples

To verify the practical applicability of the established method in industrial and regulatory scenarios, a total of 48 samples were collected for full performance validation, covering two categories: (i) pure culture reference samples of *L. paracasei* (*n* = 25); (ii) commercial probiotic products (*n* = 23), including fermented milk (*n* = 11), fermented beverages (*n* = 5), probiotic powders (*n* = 5), and dietary supplements (*n* = 2). Among the 23 commercial products, 13 were verified to contain *L. paracasei* as the functional strain, while the remaining 10 were confirmed negative for this target strain. All commercial products were purchased from local supermarkets in Nanjing, China.

All samples were tested in parallel using the established MIRA-CRISPR/Cas12a fluorescence assay and the gold-standard species-specific qPCR reference method targeting the *tuf* gene [10]. Primer sequences for the qPCR assay are listed in Table 1. The qPCR assay was performed using the ChamQ Universal SYBR qPCR Master Mix (Vazyme Biotech Co., Ltd., Nanjing, China; Cat#Q711-02) with a total reaction volume of 20 μL. The reaction system contained 10 μL 2× ChamQ SYBR qPCR Master Mix, 0.4 μL forward primer (10 μM), 0.4 μL reverse primer (10 μM), 2 μL genomic DNA template, and 7.2 μL nuclease-free water. Thermal cycling was carried out on a StepOnePlus real-time qPCR instrument (Applied Biosystems, Foster City, CA, USA) with the following program: initial denaturation at 95 °C for 5 min, followed by 40 cycles of 95 °C for 10 s and 60 °C for 30 s. Melting curve analysis was performed post-amplification to confirm the specificity of amplification products. The positive and negative results of the qPCR assay were determined with a dual-criterion threshold: samples with a cycle threshold (Ct) value ≤ 35 and a single specific melting peak consistent with the reference amplicon were defined as positive, while samples with a Ct value > 35, no amplification curve, or non-specific melting peaks were judged as negative.

### 2.9. Statistical Analysis

Statistical significance was analyzed using one-way ANOVA followed by Dunnett’s multiple comparisons test, with **** indicating *p* < 0.0001 and ns indicating no significant difference (*p* > 0.05). Quantitative image analysis procedures for specificity and sensitivity evaluations were consistent: end-point fluorescence images were captured and analyzed using ImageJ 1.54f software (National Institutes of Health, Bethesda, MD, USA). A fixed circular region of interest was uniformly applied to all reaction tubes to measure the average gray value. The relative fluorescence intensity was calculated as the average gray value of the sample divided by that of the negative control (set as 1.0). All statistical analyses were performed using GraphPad Prism 9.0 software (GraphPad Software, San Diego, CA, USA). All tests were carried out in three independent biological replicates with three technical replicates for each biological replicate. For LOD validation, the theoretical probability of capturing at least one bacterial cell in a 100 μL reaction volume was calculated using the Poisson distribution as *p* (X ≥ 1) = 1 − e^−λ^, where λ = C × V (C is the bacterial concentration in CFU/mL, and V is the sample volume in mL).

## 3. Results

### 3.1. Principle of the Visualized MIRA-CRISPR/Cas12a Detection System

**Figure 1:**
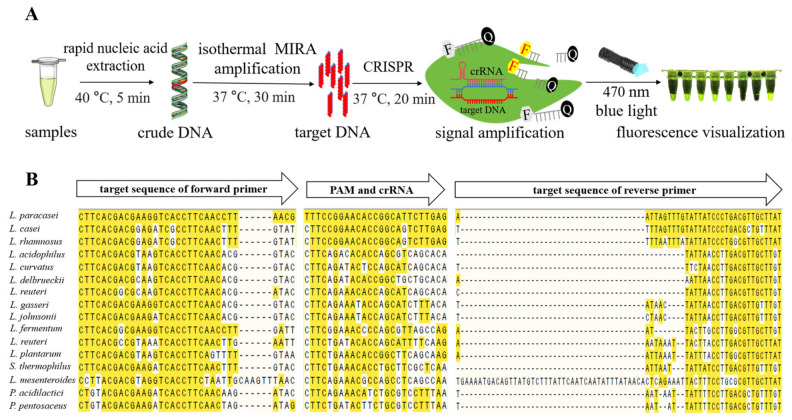
Principle and design of the MIRA-CRISPR/Cas12a detection system for *L. paracasei*. (**A**) Schematic workflow of the MIRA-CRISPR/Cas12a detection method involving four sequential steps: DNA extraction, isothermal MIRA, CRISPR/Cas12a reaction, and fluorescence visualization. (**B**) Comparative genomic alignment of the target region among diverse lactic acid bacteria, showing species-specific sequence variations in the primer-binding and crRNA target regions of *L. paracasei*.

The established *L. paracasei* detection assay integrates MIRA isothermal pre-amplification, CRISPR/Cas12a-mediated signal amplification, and visual fluorescent signal transduction, eliminating the need for professional laboratory-specific precision instruments; only basic miniaturized equipment is required (Figure 1A). Briefly, genomic DNA is rapidly extracted from samples within 8 min, followed by MIRA of the target gene within 30 min. The amplicons are then recognized by the crRNA-guided Cas12a protein during the 20 min 37 °C isothermal CRISPR reaction, triggering its non-specific trans-cleavage activity. Activated Cas12a cleaves the FAM/BHQ1-labeled ssDNA reporter, separating the fluorophore from the quencher and producing a bright green fluorescence signal that can be visualized under blue light excitation [20]. Comparative genomic alignment revealed significant interspecies sequence variations in the primer-binding sites and crRNA target locus within the conserved *rpsM* housekeeping gene between *L. paracasei* and other LAB species (Figure 1B).

**Figure 2:**
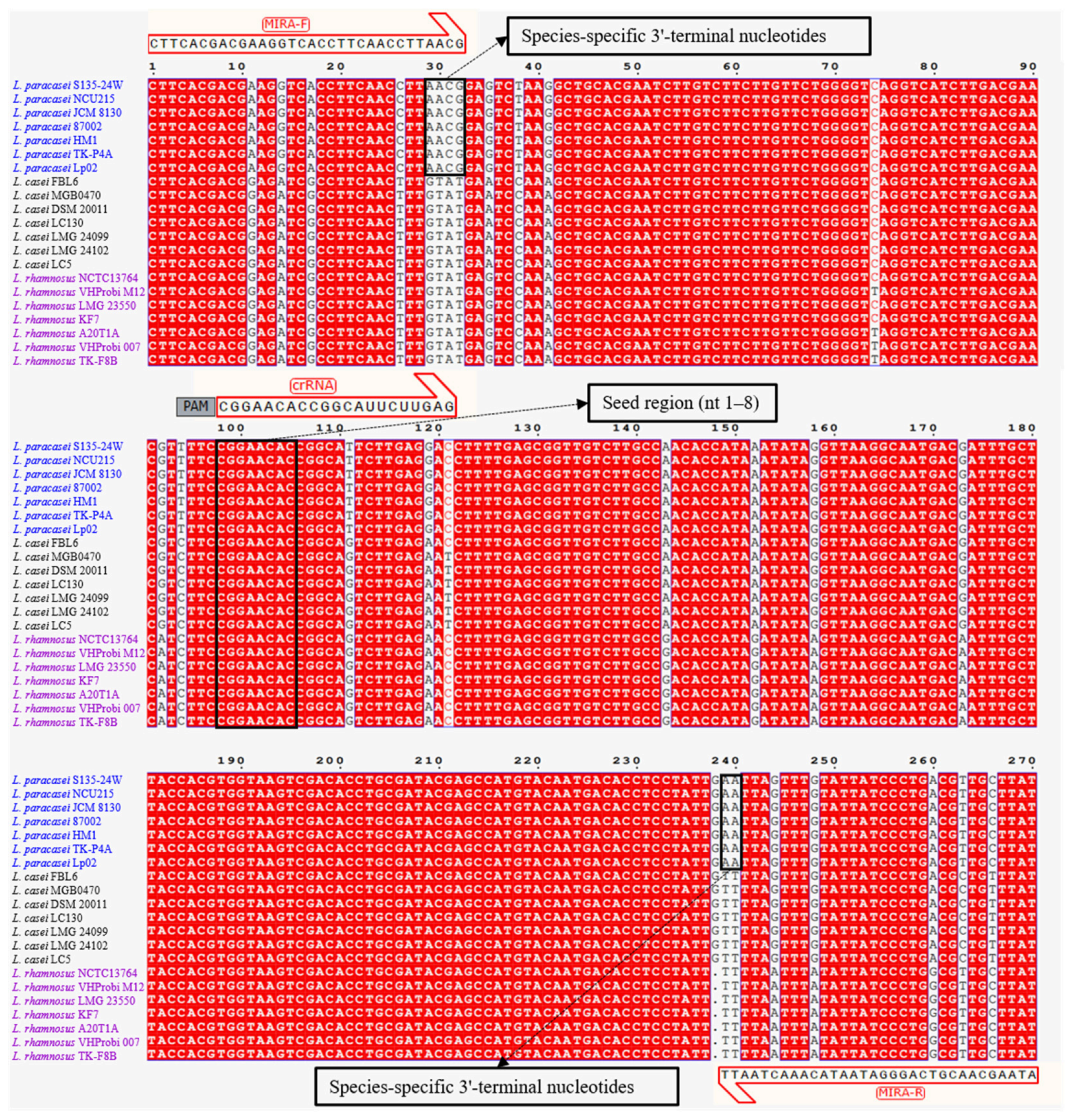
Multiple sequence alignment of the *rpsM* target region across 21 *L. casei* group strains. The alignment annotates (1) the MIRA forward and reverse primer binding sites with their 3′-terminal interspecies polymorphic sites; (2) the canonical 5′-TTTC-3′ PAM sequence in *L. paracasei* and the suboptimal 5′-CTTC-3′ PAM sequence in non-target species; (3) the crRNA target region, including the conserved seed region (nt 1–8); and (4) all interspecies nucleotide variations.

Comparative genomic alignment of 21 representative strains revealed a multi-layered discriminatory signature that enables species-specific detection despite high conservation within the *L. casei* group (Figure 2). Specifically, the 3′-termini of both MIRA primers contain interspecies polymorphic sites (forward: AACG vs. GTAT; reverse: AA vs. TT), the crRNA spacer region harbors two to three mismatches outside the seed region, and *L. paracasei* contains the canonical 5′-TTTC-3′ PAM, whereas *L. casei* and *L. rhamnosus* carry the suboptimal 5′-CTTC-3′ PAM.

### 3.2. Optimization of the CRISPR/Cas12a Reaction System

#### 3.2.1. Optimization of the Cas12a:crRNA Molar Ratio

**Figure 3:**
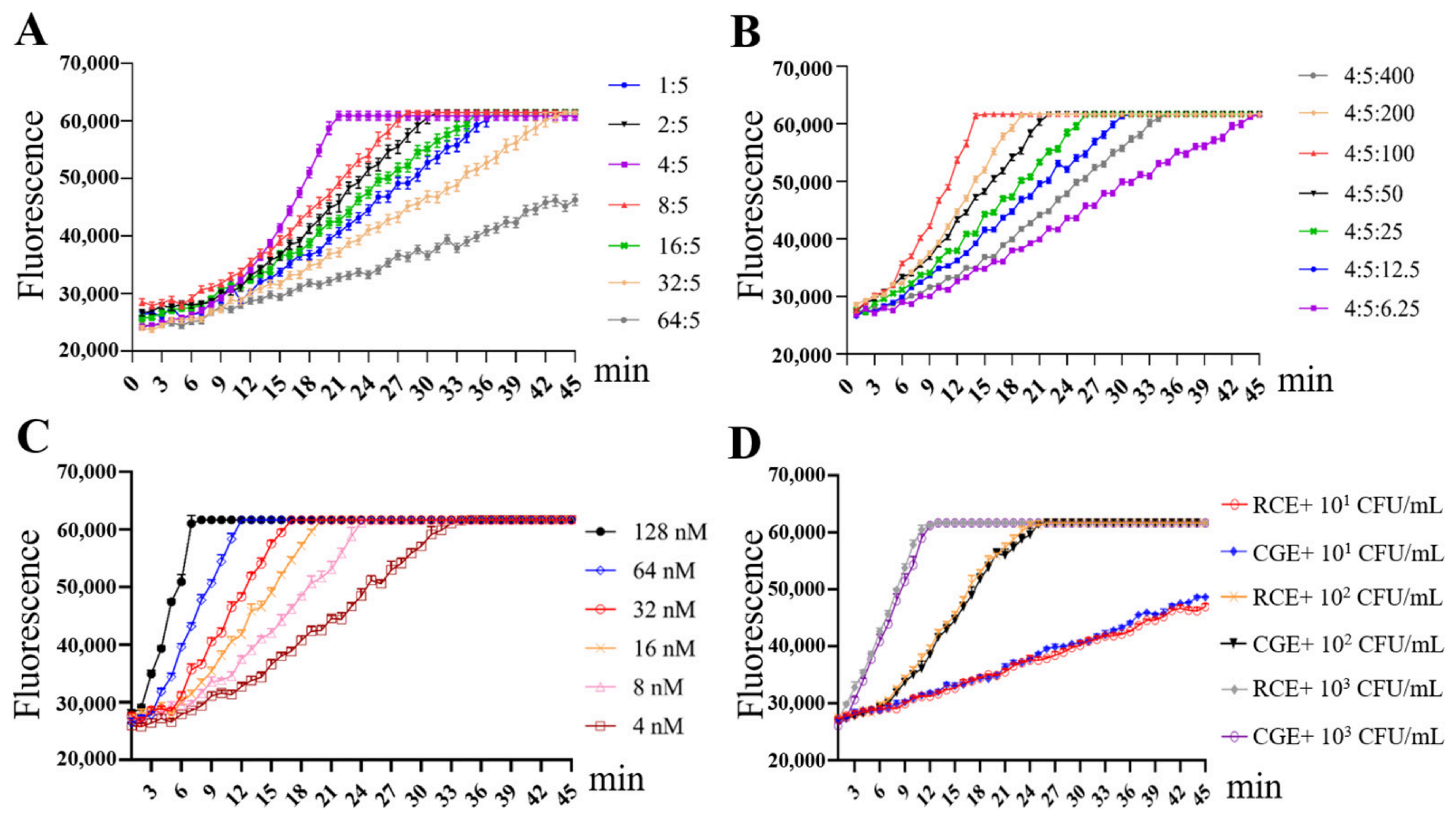
Optimization of CRISPR/Cas12a reaction conditions. (**A**) Optimization of the Cas12a:crRNA molar ratio. (**B**) Optimization of ssDNA reporter concentration. Different Cas12a:crRNA:ssDNA molar ratios (4:5:6.25, 4:5:12.5, 4:5:25, 4:5:50, 4:5:100, 4:5:200, and 4:5:400) were investigated. (**C**) Optimization of Cas12a working concentration. (**D**) Comparison of rapid crude extraction (RCE) and conventional genomic extraction (CGE) on MIRA-CRISPR/Cas12a fluorescence signals using *L. paracasei* at different concentrations.

Cas12a trans-cleavage activity dictates the sensitivity of CRISPR-based assays; thus, the core enzymatic components were optimized to improve reaction kinetics and detection performance. The Cas12a:crRNA ratio is a critical parameter for efficient RNP complex formation and subsequent trans-cleavage activation [21]. As shown in Figure 3A, a Cas12a:crRNA molar ratio of 4:5 yielded the highest initial slope (indicative of maximal trans-cleavage velocity); both suboptimal and supraoptimal ratios resulted in significantly attenuated signal output.

#### 3.2.2. Optimization of the Cas12a:crRNA:ssDNA Molar Ratio

The molar ratio of the ssDNA reporter to the pre-assembled RNP complex determines the extent of signal amplification conferred by the CRISPR reaction on MIRA products. The molar ratio of Cas12a:crRNA:ssDNA was further optimized based on the 4:5 optimal molar ratio of Cas12a:crRNA. A molar ratio of 4:5:100 (Cas12a:crRNA:ssDNA) yielded the highest initial slope (Figure 3B).

An insufficient amount of ssDNA reporter limited the rate of fluorescence accumulation, whereas excessive reporter yielded no further gain in initial reaction velocity. The latter presumably reflects the kinetic limitation of Cas12a trans-cleavage activity within the reaction window.

#### 3.2.3. Optimization of Working Concentration

Under the pre-validated optimal molar ratio of 4:5:100, we found that the fluorescence accumulation rate of the CRISPR reaction system was positively correlated with Cas12a concentration across the tested range of 4 nM to 128 nM (Figure 3C), with higher concentrations yielding faster signal accumulation. Considering the assay cost, we selected 64 nM Cas12a as the working concentration. The corresponding final working concentrations of crRNA and ssDNA were 80 nM and 1.6 μM, respectively. These concentrations correspond to the optimal molar ratio of 4:5:100 (Cas12a:crRNA:ssDNA) identified in the optimization experiments. Accordingly, the final optimized 20 μL CRISPR/Cas12a reaction system contained 5 μL MIRA-amplified product, 1× NEBuffer 2.1, 64 nM EnGen^®^ LbCas12a (NEB, Ipswich, MA, USA), 80 nM crRNA, 1.6 μM FAM-labeled ssDNA reporter, and nuclease-free water to volume. Reactions were incubated at 37 °C for 20 min, and fluorescence signals were visualized under a 470 nm blue light excitation source.

#### 3.2.4. Comparison of Two Nucleic Acid Extraction Methods

To validate the effectiveness of RCE for DNA extraction, genomic DNA of the eight LAB strains was extracted using both RCE and CGE methods for comparative analysis. Spectrophotometric purity assessment revealed that RCE products exhibited elevated A260/A280 ratios (2.07–2.32 vs. 1.76–2.01 for CGE) and markedly depressed A260/A230 ratios (0.23–0.54 vs. 0.77–2.08 for CGE) (Table S1). The elevated A260/A280 ratios may reflect RNA contamination or protein depletion, while the substantially reduced A260/A230 ratios are indicative of organic contaminants typically associated with alkaline lysis-based crude extracts. These impurities artificially inflated the apparent DNA concentrations of RCE products (359–1543 ng/μL vs. 16–141 ng/μL for CGE). Nevertheless, PCR amplification of the *16S rRNA* gene yielded the expected approximately 1500 bp bands for all samples, confirming that both methods successfully extracted amplifiable DNA from the tested strains (Figure S1).

Given the lower purity of RCE extracts, we next investigated whether such contamination compromises downstream MIRA-Cas12a detection performance. To this end, RCE and CGE were compared using *L. paracasei* samples across a range of bacterial loads. As shown in Figure 3D, comparable fluorescence signal accumulation rates were observed between the RCE and CGE groups at all tested concentrations, indicating that the rapid lysis method efficiently releases target DNA, thereby supporting effective MIRA pre-amplification and subsequent Cas12a trans-cleavage activation.

The DNA extraction efficiency of the rapid crude method is comparable to that of conventional genomic extraction, while significantly reducing the need for column purification and prolonged enzymatic digestion and completing the process in 8 min versus 2 h for CGE. These results support the integration of the RCE workflow into our detection system, enabling end-to-end rapidization and portability from nucleic acid extraction to fluorescence readout.

### 3.3. Analytical Performance of the MIRA-CRISPR/Cas12a Assay

#### 3.3.1. Specificity

**Figure 4:**
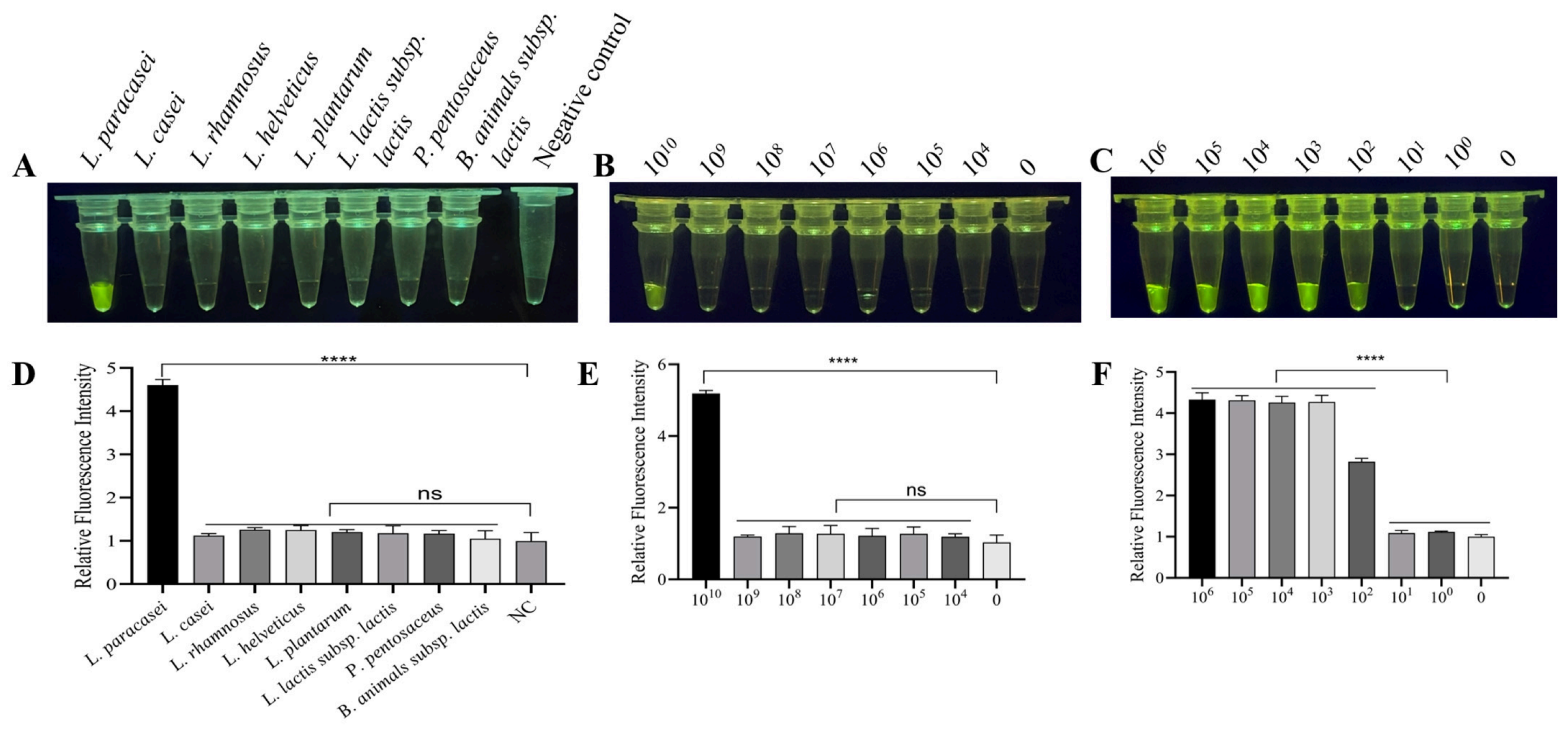
Analytical sensitivity and specificity. (**A**) Specificity evaluation of the MIRA-CRISPR/Cas12a method in different lactic acid bacteria strains. (**B**) Sensitivity analysis of the standalone CRISPR/Cas12a visual detection assay without MIRA pre-amplification. Ten-fold serially diluted *L. paracasei* spiked in sterile milk (10^4^ to 10^10^ CFU/mL) were tested. (**C**) Sensitivity analysis of the established MIRA-CRISPR/Cas12a method. Ten-fold serially diluted *L. paracasei* spiked in sterile milk (10^0^ to 10^6^ CFU/mL) were tested. (**D**) Quantitative fluorescence intensity of specificity evaluation corresponding to (**A**), analyzed by ImageJ 1.54f software. (**E**) Quantitative fluorescence intensity of standalone CRISPR/Cas12a detection corresponding to (**B**). (**F**) Quantitative fluorescence intensity of MIRA-CRISPR/Cas12a detection corresponding to (**C**). Relative fluorescence intensity was normalized to the negative control (set as 1.0). ****: *p* < 0.0001; ns: not significant (*p* > 0.05).

The analytical specificity of the established assay was evaluated in two tiers. First, a cross-genus panel of eight common food-grade LAB species spanning six genera was tested. As shown in Figure 4A, only the target *L. paracasei* generated a robust bright positive fluorescence signal, while seven non-target species, including the closely related *L. casei* and *L. rhamnosus*, exhibited no visible fluorescence and no cross-reactivity with the assay. Quantitative analysis of the fluorescence images confirmed these visual observations, with only *L. paracasei* showing a significantly elevated relative fluorescence intensity (*p* < 0.0001) and all non-target strains exhibiting signals indistinguishable from the negative control (*p* > 0.05) (Figure 4D).

Second, to rigorously validate species specificity within the taxonomically complex *L. casei* group, we expanded the validation panel to include 15 commercial probiotic strains. As shown in Table 2, all 5 *L. paracasei* strains produced strong positive fluorescence signals, while all 5 *L. casei* strains and 5 *L. rhamnosus* strains showed no detectable fluorescence. These results confirm that the assay exhibits 100% specificity for *L. paracasei* across commonly used commercial strains of *L. casei* group. The three-tier specificity mechanism ensures consistent discrimination, even when the target DNA fragment within the crRNA seed region is conserved across the genus. For the 3′-terminal, two to four nucleotides of both MIRA primers are strictly conserved in all *L. paracasei* strains but harbor mismatches in *L. casei* and *L. rhamnosus*, thereby completely blocking recombinase-mediated primer extension and preventing non-specific amplification [16,22]. Furthermore, Cas12a requires PAM binding to initiate DNA unwinding; compared with the suboptimal 5′-CTTC-3′ PAM in *L. casei* and *L. rhamnosus*, the canonical 5′-TTTC-3′ PAM in *L. paracasei* activates Cas12a with 1.4- to 5-fold higher cleavage efficiency on average [23]. The 2–3 bp mismatches in the crRNA target region of *L. casei* and *L. rhamnosus* relative to the target sequence also attenuate Cas12a trans-cleavage activity [24]. Combined with the 10-fold sample dilution in the amplification system and the intrinsic LOD of the CRISPR reaction (Figure 4B), we speculate that cross-reactivity might only be observed at bacterial concentrations exceeding 10^12^ CFU/mL—a range typical of frozen and concentrated starter culture raw materials, not commercial finished food products [7,25].

#### 3.3.2. Sensitivity

Isothermal pre-amplification is an indispensable prerequisite for CRISPR/Cas12a-based nucleic acid detection, as standalone CRISPR/Cas12a systems suffer from an inherent analytical sensitivity limitation. Although this limitation is well recognized, we first evaluated the feasibility of direct CRISPR/Cas12a detection without pre-amplification. Commercial probiotic foods typically contain viable LAB cells at concentrations ranging from 10^6^ CFU/mL (regulatory minimum) to 10^9^ CFU/mL (high-content formulations), which we hypothesized might approach the detection threshold of unamplified CRISPR systems. However, only the 10^10^ CFU/mL sample yielded distinct green fluorescence (Figure 4B). Quantitative image analysis confirmed this result, with only the 10^10^ CFU/mL sample showing a measurable fluorescence signal above background (Figure 4E).

To address this limitation, we coupled isothermal MIRA pre-amplification with CRISPR/Cas12a signal amplification. This integrated assay achieved a reliable LOD of 100 CFU/mL for *L. paracasei* in milk-matrix spiked samples, with visually detectable green fluorescence (Figure 4C,F). This sensitivity meets practical requirements across the probiotic food chain: routine verification of products at or above the 10^6^ CFU/mL regulatory minimum, monitoring of strain decline below labeled claims in multi-strain formulations or near end-of-shelf-life, and detection of low-level unintended contamination during manufacturing. Such performance is critical for end-to-end quality control and regulatory compliance.

This result is consistent with the theoretical prediction of the Poisson distribution (Section 2.9). For the 100 μL sample volume, the expected probability of capturing at least one cell is ~63.2% at 10 CFU/mL (λ = 1.0), ~9.5% at 1 CFU/mL (λ = 0.1), and ~1.0% at 0.1 CFU/mL (λ = 0.01). The observed 44.4% positive rate at 10 CFU/mL reflects expected stochastic variation, while the absence of positives at ≤1 CFU/mL aligns with the low theoretical probabilities (Table 3). Therefore, the inconsistent results below the reliable LOD of 100 CFU/mL are attributable to inherent statistical sampling limitations rather than detection method insufficiency.

The sensitivity of MIRA-CRISPR is comparable to that of RPA-CRISPR/Cas12a but superior to that of LAMP-CRISPR/Cas12a. RPA-Cas12a assays have achieved LODs as low as 1 CFU/mL for *Salmonella* in spiked beer and juice samples, 10^2^ CFU/mL for *Salmonella* in egg and chicken products, and 1 CFU/mL for *Diaporthe aspalathi* using target gene plasmid standards [15,26,27]. LAMP-Cas12a assays have achieved an LOD of 10 copies/reaction (equivalent to 4000 copies/mL plasmid standard) for *Candidatus Liberibacter asiaticus* in citrus leaves and 10^4^ copies/mL SARS-CoV-2 in nasopharyngeal swabs [26,28].

### 3.4. Comparative Evaluation of Real Food Sample Detection

To further verify the practical applicability of the established assay, a total of 48 samples were collected for full performance validation, comprising two core categories: (i) pure culture reference samples with gradient bacterial loads of *L. paracasei* (*n* = 25, serving as standard quality control samples); (ii) commercial probiotic products (*n* = 23), including fermented milk (*n* = 11), fermented beverages (*n* = 5), probiotic powders (*n* = 5), and probiotic dietary supplements (*n* = 2).

**Figure 5:**
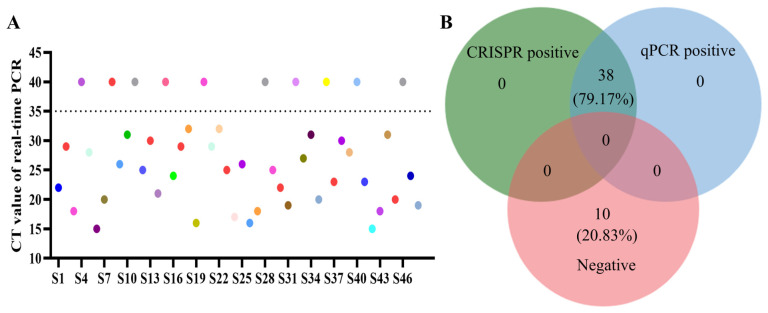
Performance evaluation of the MIRA-CRISPR/Cas12a method for real food sample detection. (**A**) Cycle threshold (Ct) values for 48 food samples detected by the qPCR reference method. (**B**) Venn diagram showing the detection concordance between the MIRA-CRISPR/Cas12a assay and the qPCR reference method.

The results showed 100% diagnostic concordance between the MIRA-CRISPR/Cas12a assay and the validated species-specific qPCR reference method: 25 pure culture reference samples and 13 commercial products tested positive by both methods, and 10 commercial products were negative (Figure 5B). The qPCR cycle threshold (Ct) values for positive samples ranged from 15 to 33 (Figure 5A), corresponding to a wide linear range of bacterial loads from high-concentration pure cultures to low-abundance commercial products. The MIRA-CRISPR/Cas12a assay successfully detected all positive samples across the entire Ct range, exhibiting stable and reliable detection performance regardless of target concentration or food matrix type, including liquid products (e.g., fermented milk) and solid products (e.g., probiotic powders). No false-positive or false-negative results were observed in this validation test, which further confirmed that the high specificity and high sensitivity of the established assay verified in Section 3.3 can be reproduced in complex real food samples.

## 4. Discussion

The newly developed MIRA-CRISPR/Cas12a detection platform offers multiple advantages over existing methods for *L. paracasei* identification (Table 4). Relative to conventional culture-based biochemical identification, our assay achieves markedly reduced turnaround time and species-level specificity, compressing detection from 48 to 72 h to 58 min (8 min nucleic acid extraction, 30 min MIRA, and 20 min CRISPR reaction) [29]. Versus PCR-based methods, including qPCR and droplet digital PCR, our method eliminates the requirement for bulky, non-portable, and expensive thermal cycling equipment, rendering it suitable for resource-limited settings [30,31]. The direct reagent cost per test is below $5, and the total cost per test (including instrument amortization) is lower than that of qPCR and droplet digital PCR. Sensitivity is comparable to that of most reported qPCR assays, though inferior to that of droplet digital PCR, which remains highly dependent on professional and expensive instruments [10,12,31,32].

Subsequent work will focus on lyophilized reagent formulation for streamlined operation, coupled with a fully closed-tube integrated system (single-tube sequential or one-pot simultaneous reaction) to eliminate aerosol contamination risks. Ultimately, miniaturized supporting instruments and matched consumables will be integrated into an all-in-one portable on-site detection kit, unlocking wide application prospects for rapid quality screening of probiotic products across production workshops and frontline regulatory scenarios.

## 5. Conclusions

In this study, we developed a fluorescence-visualized, field-deployable MIRA-CRISPR/Cas12a detection method for rapid, species-specific, and highly sensitive identification of *L. paracasei* in LAB-fortified food products. This was achieved through the following: (i) innovative coupling of a signal cascade amplification (MIRA pre-amplification and CRISPR/Cas12a trans-cleavage); (ii) establishment of a rapid alkaline lysis-based DNA extraction protocol; (iii) rational design of species-specific primers and crRNA; and (iv) systematic optimization of key reaction parameters.

## Figures and Tables

**Table 1:** Sequence of primers and crRNA used in this study.

Name	Sequence (5′-3′)	Amplicons (bp)	Target Gene
MIRA-F	CTTCACGACGAAGGTCACCTTCAACCTTAACG	140	*rpsM*
MIRA-R	ATAAGCAACGTCAGGGATAATACAAACTAATT		
crRNA	UAAUUUCUACUAAGUGUAGAUCGGAACACCGGCAUUCUUGAG	/	*rpsM*
qPCR-F	TCCGGGAACTGCTCAGC	161	*tuf*
qPCR-R	TGTTTCACGAACAGGTG		
ssDNA	FAM-NNNNNN-BHQ1	/	/
16SF	AGAGTTTGATCCTGGCTCAG	1484	*16s* *rRNA*
1495R	CTACGGCTACCTTGTTACGA		

**Table 2:** Specificity validation of the MIRA-CRISPR/Cas12a assay across commercial *L. casei* group strains.

Species	Strain Designation	Assay Result
*Lacticaseibacillus paracasei*	GM080	+
	GMNL-33	+
	LcS	+
	K56	+
	LC01	+
*Lacticaseibacillus casei*	BD-8632	−
	B63	−
	B240	−
	ATCC 393	−
	LC89	−
*Lacticaseibacillus rhamnosus*	GG (LGG^®^)	−
	HN001	−
	MP108	−
	GR-1	−
	ATCC 53103	−

**Table 3:** Positive rate of the MIRA-CRISPR/Cas12a assay at different bacterial concentrations.

Concentration (CFU/mL)	Positive/Total Replicates	Positive Rate (%)
1 × 10^6^	9/9	100
1 × 10^5^	9/9	100
1 × 10^4^	9/9	100
1 × 10^3^	9/9	100
1 × 10^2^	9/9	100
1 × 10^1^	4/9	44.4
1 × 10^0^	0/9	0

**Table 4:** Comparison of the established MIRA-CRISPR/Cas12a detection assay with existing methods for *L. paracasei* identification.

Characteristic	Biochemical Identification	QPCR	Droplet Digital PCR	MIRA-CRISPR/Cas12a (This Study)
Specificity	Genus-level, cross-reactivity common	Species-level, no cross-reactivity	Species-level, no cross-reactivity	Species-level, no cross-reactivity
Sensitivity	10^2^–10^3^ CFU/mL	10^2^–10^3^ CFU/mL	10^0^–10^1^ CFU/mL	10^2^ CFU/mL
Total detection time	48–72 h	2–4 h	4–6 h	58 min
Cost per test	$2–3	$2–3	$4–6	$4–5
Specialized equipment	Biochemical ID system	Real-time thermal cycler	Droplet digital PCR system	Blue light exciter
Skill requirement	Basic	Molecular expertise	Advanced expertise	Basic
Naked-eye visualization	Colony morphology	Instrument-only readout	Instrument-only readout	Blue-light readout
On-site applicability	Not applicable	Not applicable	Not applicable	applicable

## Data Availability

All data generated or analyzed during this study are included in this published article. The raw data supporting the conclusions will be made available by the authors without undue reservation to any qualified researcher.

## Supplementary Materials

The following supporting information can be downloaded at https://www.mdpi.com/article/10.3390/foods15152709/s1. Figure S1: Validation of DNA extraction integrity by universal bacterial 16S rRNA gene PCR; Table S1: Comparison of DNA purity and concentration extracted by RCE and CGE methods from different lactic acid bacteria strains.

## Abbreviations

The following abbreviations are used in this manuscript:

qPCR	quantitative Real-Time Polymerase Chain Reaction
MIRA	Multiple cross displacement amplification
CRISPR	Clustered Regularly Interspaced Short Palindromic Repeats
Cas12a	CRISPR-associated protein 12a
ssDNA	single-stranded Deoxyribonucleic Acid
LAB	Lactic Acid Bacteria
CICC	China Center of Industrial Culture Collection
MRS	De Man–Rogosa–Sharpe
PBS	Phosphate-Buffered Saline
bp	base pair
nt	nucleotide
PAM	Protospacer-Adjacent Motif
FAM	6-carboxyfluorescein
HPLC	High-Performance Liquid Chromatography
BHQ1	Black Hole Quencher 1
RNP	Ribonucleoprotein
CFU	Colony-Forming Unit
LOD	Limit of Detection
Ct	Cycle Threshold
