# Supplementary material for: Rapid On-Site Detection of Lacticaseibacillus paracasei Using a Portable MIRA-CRISPR/Cas12a Naked-Eye Fluorescence Assay
Source: Foods. 2026 Jul 31;15(15):2709. doi: 10.3390/foods15152709 (PMC13464588; doi:10.3390/foods15152709)
Supplement: Supplementary file 1 [file foods-15-02709-s001.zip › Supplementary Files/Table S1.pdf]

Table S1. Comparison of DNA purity and concentration extracted by RCE and CGE methods from different lactic acid bacteria strains.

Strain	Method	A260/A280	A260/A230	Conc. (ng/μL)
P. pentosaceus	RCE	2.08 ± 0.19	0.34 ± 0.14	680.26 ± 82.9
	CGE	1.77 ± 0.12	0.77 ± 0.09	140.95 ± 11.8
L. helveticus	RCE	2.14 ± 0.19	0.54 ± 0.10	1542.55 ± 199.9
	CGE	1.90 ± 0.16	2.08 ± 0.11	34.68 ± 6.3
L. paracasei	RCE	2.18 ± 0.12	0.23 ± 0.11	359.73 ± 42.0
	CGE	1.76 ± 0.14	1.32 ± 0.07	16.33 ± 6.0
B. animalis subsp. *lactis*	RCE	2.07 ± 0.13	0.24 ± 0.12	432.07 ± 48.4
	CGE	1.91 ± 0.18	1.42 ± 0.07	22.97 ± 9.1
L. rhamnosus	RCE	2.15 ± 0.15	0.48 ± 0.12	721.75 ± 61.0
	CGE	2.01 ± 0.19	1.56 ± 0.11	64.90 ± 8.9
L. plantarum	RCE	2.32 ± 0.13	0.26 ± 0.10	476.87 ± 61.0
	CGE	1.93 ± 0.14	1.47 ± 0.07	47.61 ± 5.2
L. lactis subsp. lactis	CGE	2.19 ± 0.19	0.27 ± 0.18	564.00 ± 71.3
	RCE	1.85 ± 0.13	1.28 ± 0.09	72.00 ± 8.5
L. casei	CGE	2.08 ± 0.18	0.39 ± 0.14	619.00 ± 90.2
	RCE	1.80 ± 0.19	1.42 ± 0.09	23.00 ± 5.6

Note: Data are presented as mean ± standard deviation ($\overline{x}$ ± SD), n = 3.
